# Use of the Internet for Health Purposes—A National Web-Based Cross-Sectional Survey among Adults in Poland

**DOI:** 10.3390/ijerph192316315

**Published:** 2022-12-06

**Authors:** Krzysztof Płaciszewski, Waldemar Wierzba, Janusz Ostrowski, Jarosław Pinkas, Mateusz Jankowski

**Affiliations:** 1 Central Clinical Hospital of the Ministry of the Interior and Administration in Warsaw, 02-507 Warsaw, Poland; 2 School of Public Health, Centre of Postgraduate Medical Education, 01-826 Warsaw, Poland

**Keywords:** eHealth, internet use, online health information, internet use for health purposes, health behaviors, health information, Poland

## Abstract

The Internet is one of the most popular information sources. This study aimed to assess the public attitudes towards the use of the Internet for health purposes as well as to identify factors associated with the use of the Internet for health purposes among adults in Poland. A web-based cross-sectional survey was carried out between 9 and 12 September 2022 on a nationwide random-quota sample of 1092 adults in Poland. The study questionnaire included 10 questions on Internet use for health purposes. The most common reason for the use of the Internet for health purposes was searching for information on drugs and their effects (69.9%). Almost two-thirds of participants used the Internet for searching for health information (64.9%), for doctors/medical services (63.4%), or for medical facilities (65.3%). Over half of the participants used the Internet for checking online reviews of doctors (55.2%) and 43.5% of the participants ordered drugs or dietary supplements online. Out of 9 different socioeconomic factors analyzed in this study, having higher education, being female, as well as living in cities from 100,000 to 499,999 residents were the most important factors (*p* < 0.05) associated with the use of the Internet for health purposes. This study confirmed a high level of adoption of medical Internet in Poland.

## 1. Introduction

The Internet is one of the most popular information sources [1]. It is estimated that two-thirds of the global population has an Internet connection, wherein the highest number of Internet users is observed in the group aged 25–34 years [1]. The global internet penetration rates vary from 98% in Northern Europe to 25% in Middle Africa [2]. In the European Union, over 90% of households have Internet access [2]. Mobile Internet is responsible for 60% of total web traffic [1,2].

Socioeconomic changes evoked by the widespread Internet access also affected healthcare and health systems. Health and well-being content is widely available on major news websites around the world [3,4]. Moreover, social media pose a significant source of health-related information [5]. Web-based discussion forums, especially those dedicated to chronic or rare diseases, are also gaining popularity [6]. Medical Internet provides easy access to information on diseases symptoms, drug action, and information on how to proceed in the event of suspected disease [7]. However, there is a relatively high prevalence of health misinformation on the Internet (e.g., on vaccinations), increased particularly through social media [8]. National public health agencies and international health organizations are publishing evidence-based health information that is open-access and free of charge for the public [9,10]. Nevertheless, the growing number of medical misinformation on the Internet is a significant public health issue. The remarkable development of the e-commerce market also had an impact on healthcare and health services. A growing number of medical facilities and health organizations, on their social media profiles, inform users about their services and enable online registration [11]. Moreover, online pharmacies and medical stores that offer mail-order sales of drugs and healthcare supplies are gaining popularity [12].

The widespread implementation of the Internet in healthcare has led to the development of a new healthcare branch—eHealth [13]. In general, eHealth can be defined as “health services and information delivered or enhanced through the Internet and related technologies” [13]. The most common implementation of eHealth technologies into national health systems are teleconsultations, e-prescriptions, electronic health records, and other related health services [14]. Moreover, eHealth interventions are used to promote a healthy lifestyle in the general population or pro-healthy attitudes among patients with chronic diseases [15,16]. However, the use of eHealth services depends on an individual’s eHealth literacy levels [17]. eHealth literacy is defined as a set of skills, competencies, and attitudes related to accessing, understanding, appraising, and applying health-related information available from electronic sources [17].

The COVID-19 pandemic has had an important impact on the development of eHealth services as well as the global use of the Internet for health purposes [18]. In many countries, anti-epidemic measures limited access to health services, which was compensated by the development of telehealth services [19,20]. During the COVID-19 pandemic, the importance of the Internet as a source of health information has significantly increased.

Between 2007 and 2017, in the European Union (EU), the percentage of households with Internet access increased from approximately 55% to 87% [21]. In 2014, over 75% of EU citizens declared that the Internet is a good way of finding out more about health [21]. Findings from the Flash Eurobarometer No. 404 (2014) showed significant differences in the use of the Internet for health information according to gender, age, education, long-term illness, and health-related knowledge [21]. An increase in the number of EU households with Internet access may lead to higher medical internet utilization. In recent years, a growing number of health-related content is published via social media, especially Facebook, Instagram, and TikTok [5].

Poland is a European country where over 32 million citizens declare Internet use (approximately 85% of the population) [22]. Moreover, there is widespread access to public eHealth services such as e-prescription, electronic sick leave, teleconsultation, and individual mobile health records (Internet Patients’ Account) available through a dedicated web portal [23]. Public health authorities in Poland are active on social media and regularly publish health-related content. Moreover, private healthcare providers use eHealth, for example, by offering access to mobile health applications, a virtual doctor’s office, or an e-schedule that allows the patient to make an appointment in a stationary or online medical facility [24].

Economic growth and social changes observed in Poland during the past three decades may have a significant impact on public attitudes toward health choices, health-related behaviors, and the use of healthcare services [25]. Trends in the use of the Internet for health purposes in Poland were regularly monitored by public opinion research agencies [26]. Between 2006 and 2012, the percentage of inhabitants of Poland who used the Internet for health-related purposes increased from 41.7% to 66.7% [26]. Despite the study years, the highest percentage of Internet users were in the youngest age groups [26]. Moreover, over one-quarter of adults in Poland declared the use of interactive Internet health services, such as communicating online with a health care provider [26]. In January 2018, the prevalence of Internet use for health purposes was estimated at 76.9% of the adult population in Poland [27].

There is a lack of up-to-date data on the use of the Internet for health purposes in Poland after the COVID-19 pandemic. Regular monitoring of the use of the Internet for health purposes may inform public health authorities about further organizational and educational needs related to the medical Internet and eHealth use. Moreover, changes in patterns of Internet use for health purposes may inform policymakers about the regulation needs and the trends in medical Internet use in Poland, which may be used by public health institutions to develop educational campaigns and interventions. Data on the use of the Internet for health purposes among adults in Poland may be a benchmark for developing countries that are facing similar socioeconomic changes as Poland during the past 20 years.

Therefore, this study aimed to assess the use of the Internet for health purposes as well as to identify factors associated with the use of the Internet for health purposes among adults in Poland.

## 2. Materials and Methods

### 2.1. Study Design and Population

Data were collected between 9 and 12 September 2022. A nationwide random-quota sample of 1092 participants was selected from >100,000 adults registered in the Nationwide Research Panel Ariadna—the public opinion survey company operating in Poland [28]. The stratification model included gender, age, and place of residence. A similar approach was used in previously published studies on public attitudes toward vaccines or tobacco use in Poland [29,30]. Data used for sample size calculation and stratification were driven from the public registry managed by Statistics Poland. The study sample is representative of adult Internet users in Poland [28,29,30]. It is estimated that 92.4% of households in Poland have Internet access [31]. The study questionnaire was available through the dedicated web portal (computer-assisted web interview—CAWI) managed by the survey company. Datasets generated during this study were anonymous.

### 2.2. Questionnaire

The study questionnaire was prepared for the purposes of this publication and included 10 questions on Internet use for health purposes. During the preparation of the questionnaire, a literature review (PubMed database; papers on the use of the Internet for health purposes in a representative sample of adults, published between 2015 and 2022, were analyzed) was performed to identify current trends in the use of the Internet for health purposes in Poland [26,27] and the Health Information National Trends Survey (HINTS) was consulted [32]. Ten different reasons for the use of the Internet for health purposes during the last 12 months were analyzed using the following question: “During the last 12 months did you use the Internet for the following purposes: (1) searching for health information; (2) searching for information about doctors and medical services; (3) searching for a medical facility; (4) checking online reviews of doctors; (5) checking online reviews of the medical facility; (6) making an appointment with a doctor; (7) checking the results of medical/laboratory tests; (8) searching for information on drugs and their effects; (9) ordering a prescription for drugs via the Internet; (10) purchase of drugs or dietary supplements via the Internet; single-choice answer: yes/no”. Moreover, a set of questions on socioeconomic characteristics was addressed. Higher educational level was defined as having a university (master’s or bachelor’s) degree. A pilot study was conducted; 10 adults (aged 18–62 years) filled out the questionnaire twice, 5 days apart. After the analysis of the responses, two questions were rewritten to clarify the text.

### 2.3. Statistical Analysis

Data were analyzed with the IBM SPSS software version 28. Frequencies and proportions were used to present distributions of categorical variables. Chi-squared test with cross-tabulations was used to compare categorical variables.

Associations between nine socioeconomic factors (independent variables: gender, age, education, marital status, having children, place of residents, number of household members, employment status, and self-declared economic status) and the use of the Internet for particular health purposes (dependent variable) were analyzed using multivariable logistic regression models. The strength of association was presented by the odds ratio (OR) along with 95% confidence intervals (95% CI). The level of statistical significance was set at *p* < 0.05.

### 2.4. Ethics

Participation in this study was voluntary. The study protocol was approved by the Ethics Review Board at the Central Clinical Hospital of the Ministry of the Interior and Administration in Warsaw, Poland (approval number 41/2022).

## 3. Results

### 3.1. Study Population

The study population included 1092 adults, aged 18–88 years; 52.6% of participants were females (Table 1). The socioeconomic characteristics of the study population are presented in Table 1.

### 3.2. The Use of the Internet for Health Purposes

The most common reason for the use of the Internet for health purposes was searching for information on drugs and their effects (69.9%). Almost two-thirds of participants used the Internet for searching for health information (64.9%), searching for information about doctors and medical services (63.4%), or searching for a medical facility (65.3%). Over half of the participants used the Internet for checking online reviews of doctors (55.2%), checking online reviews of the medical facility (51.5%), or checking the results of medical/laboratory tests (52.0%). Less than half of the participants used the Internet for making an appointment with a doctor (43.8%) or to purchase drugs or dietary supplements (43.5%). The least popular form of the use of the Internet for health purposes (27.7%) was ordering a prescription for drugs via the Internet (Table 2).

There were significant differences in the percentage of participants who used the Internet for particular health purposes by socioeconomic variables (Table 2). In general, females more often declared the use of the Internet for health purposes. The percentage of participants who used the Internet for health purposes was the lowest among those who lived in the smallest cities (<20,000 residents). Moreover, age differences in the use of the Internet for health purposes were observed. There were no significant differences in the percentage of participants who used the Internet for particular health purposes, except for checking the results of medical/laboratory tests (*p* = 0.02).

### 3.3. Factors Associated with the Use of the Internet for Health Purposes

The impact of nine different variables on the use of the Internet for health purposes was assessed using multivariable logistic regression models (Table 3). Females (OR: 1.82; 95% CI: 1.39–2.37; *p* < 0.001), those with higher education (OR: 1:38; 95% CI: 1.06–1.81; *p* = 0.02), as well as those who lived in cities from 100,000 to 499,999 residents (OR: 1.49; 95% CI: 1.02–2.18; *p* = 0.04) were more likely to declare that they used the Internet for searching for health information. Females (OR: 1.47; 95% CI: 1.13–1.92; *p* = 0.004), participants under 50 years of age (*p* < 0.05), those with higher education (OR: 1.55; 95% CI: 1.18–2.03; *p* = 0.002), participants who had children (OR: 1.51; 95% CI: 1.01–2.28; *p* = 0.04), as well as those who lived in cities from 100,000 to 499,999 residents (OR: 1.82; 95% CI: 1.23–2.70; *p* = 0.003) were more likely to declare that they used the Internet for searching for information about doctors and medical services. Females (OR: 1.47; 95% CI: 1.12–1.92; *p* = 0.005), participants under 50 years of age (*p* < 0.05), those with higher education (OR: 1.49; 95% CI: 1.14–1.96; *p* = 0.004), participants who had ever been married (OR: 1.50; 95% CI: 1.05–2.13; *p* = 0.03), as well as those who lived in cities from 100,000 to 499,999 residents (OR: 1.96; 95% CI: 1.31–2.91; *p* < 0.001) were more likely to declare that they used the Internet for searching for a medical facility. Females (OR: 1.83; 95% CI: 1.41–2.37; *p* < 0.001), participants under 50 years of age (*p* < 0.05), those with higher education (OR: 1.52; 95% CI: 1.17–1.98; *p* = 0.002), as well as those who lived in cities from 100,000 to 499,999 residents (OR: 2.05; 95% CI: 1.41–2.99; *p* < 0.001) were more likely to declare that they used the Internet for checking online reviews of doctors. Females (OR: 1.46; 95% CI: 1.46–1.89; *p* = 0.003), participants under 40 years of age (*p* < 0.05), those who had children (OR: 1.57; 95% CI: 1.05–2.34; *p* = 0.03), as well as those who lived in cities from 20,000 to 499,999 residents (*p* < 0.05) were more likely to declare that they used the Internet for checking online reviews of the medical facility. Participants aged 18–29 years (OR: 2.09; 95% CI: 1.23–3.54; *p* = 0.01), those with higher education (OR: 1.68; 95% CI: 1.30–2.18; *p* < 0.001), those who lived in cities above 99,999 residents (*p* < 0.05), as well as those who lived with at least two persons in one household (OR: 1.58; 95% CI: 1.01–2.48; *p* = 0.048) were more likely to declare that they used the Internet for making an appointment with a doctor. Participants who had higher education (OR: 1.86; 95% CI: 1.43–2.41; *p* < 0.001), those who lived in cities above 99,999 residents (*p* < 0.05), those who lived with at least two persons in one household (OR: 2.03; 95% CI: 1.30–3.16; *p* = 0.002), those who were employed/self-employed (OR: 1.36; 95CI: 1.01–1.83; *p* = 0.04), as well as those participants who declared good economic status (OR: 1.42; 95% CI: 1.01–2.00; *p* = 0.04) were more likely to declare that they used the Internet for checking the results of medical/laboratory tests. Females (OR: 2.11; 95% CI: 1.60–2.80; *p* < 0.001), those with higher education (OR: 1.53; 95% CI: 1.14–2.03; *p* = 0.004), those who had children (OR: 1.60; 95% CI: 1.03–2.48; *p* = 0.04), those who lived in cities above 99,999 residents (*p* < 0.05), as well as those who lived with at least two persons in one household (OR: 1.64; 95% CI: 1.03–2.61; *p* = 0.04) were more likely to declare that they used the Internet for searching for information on drugs and their effects. Participants who had higher education (OR: 1.33; 95% CI: 1.01–1.77; *p* = 0.046) and those who lived in cities above 99,999 residents (*p* < 0.05) were more likely to declare that they used the Internet for ordering a prescription for drugs. Females (OR: 1.41; 95% CI: 1.09–1.82; *p* = 0.01), those with higher education (OR: 1.58; 95% CI: 1.22–2.05; *p* < 0.001), those who lived in cities above 99,999 residents (*p* < 0.05), as well as those who lived with at least two persons in one household (OR: 1.68; 95% CI: 1.08–2.60; *p* = 0.02), were more likely to declare that they used the Internet for the purchase of drugs or dietary supplements (Table 3).

## 4. Discussion

In this study, a comprehensive characteristic of the use of the Internet for health purposes was presented. Out of 10 different reasons for the use of the medical Internet, searching for information on drugs and their effects, searching for health information, searching for information about doctors and medical services, and searching for a medical facility were the most common responses declared by the participants. Out of 9 different socioeconomic factors analyzed in this study, having higher education, being female, as well as living in cities from 100,000 to 499,999 residents were the most important factors associated with the use of the Internet for health purposes.

The Internet offers easy access to health-related information [33]. Publications on the Internet reach a wide audience, with low effort and resources on the part of the publisher, which makes it one of the most cost-effective methods of health communication [34]. In this study, 64.9% of adults in Poland used the Internet for searching for health information and 69.9% used the Internet for searching for information on drugs and their effects, which is lower than reported by Bujnowska-Fedak et al. in 2018 (76.9% of participants used the Internet as a source of health information and services) [27]. We can hypothesize that differences between our study and the study by Bujnowska-Fedak et al. [27] may result from different methodologies (in this study, Internet use for health purposes in the last 12 months was assessed vs. use in general in the study by Bujnowska-Fedak et al.) and populations (this study was carried out among active Internet users with the CAWI technique vs. computer-assisted telephone interview in a general population in the study by Bujnowska-Fedak et al.) [27]. Moreover, we can hypothesize that the spread of health misinformation on the Internet increased during the COVID-19 pandemic, which may decrease the levels of public trust in the medical information available on the Internet [8]. Bujnowska-Fedak et al. reported that in 2018, most individuals searching for health information on the Internet had higher education, lived in urban areas, and were occupationally active [27]. In this study, higher education was also significantly associated with the use of the Internet for searching for health information, but there were no significant differences depending on age and occupational status. We can hypothesize that the Internet is currently widely available in Poland [22], so there is a lack of economic barriers (e.g., by occupational or financial status) to Internet access. This observation is in line with the study by Żarnowski et al., who reported a lack of socioeconomic barriers in the use of mobile apps and wearables to monitor diet, weight, and physical activity among adults in Poland in 2022 [35]. Moreover, there are numerous activities targeted at older adults that tend to increase the digital competencies of the elderly and remove age-related barriers to Internet use.

Healthcare service providers and medical facilities are often present on the Internet and social media [36]. Moreover, a growing number of healthcare professionals have their professional profiles on Facebook or Instagram to inform potential patients about their competence, services, and the workplace [37]. In Poland, most medical facilities have their website. Moreover, online platforms with reviews of doctors and medical facilities are also easily available. Findings from this study showed that almost two-thirds of participants had searched for information on doctors and medical facilities and over half of the participants had checked the online reviews of doctors and medical facilities. This finding points out that Poles pay more and more attention to the quality of medical services. The checking of online reviews of doctors is particularly common in the case of surgeons and private healthcare services [38]. We can hypothesize that patients check the opinion of doctors that they have not previously met, but online communication with doctors mostly refers to visits related to the continuation of the treatment rather than the first visit. In this study, female gender, age under 50 years, having higher education, and living in cities from 100,000 to 499,999 residents were the most important factors associated with the online search for doctors, medical services, medical facilities, and checking their reviews. We can hypothesize that younger adults (<50 years) have a better health status and a lower number of health problems, so when the first health problem occurs, they tend to find the most suitable doctor/medical facility to solve this health problem. Moreover, medium-sized cities (100,000–499,999 citizens) offer a wider choice of medical facilities than rural areas or small cities and relatively shorter waiting times than in the largest cities. Gender differences in the use of the Internet for health purposes may result from the fact that females place more attention on health-related issues [39].

One of the most common eHealth services available in Poland is the possibility of online registration for a medical appointment [23]. During the COVID-19 pandemic, each citizen of Poland has an opportunity to schedule a vaccination appointment via a dedicated IT system (Internet Patients’ Account) [23]. In this study, 43.8% of participants declared that they used the Internet for scheduling medical appointments. Online registration was the most common among the youngest participants (18–29 years), those with higher education, those who lived in cities above 100,000 residents, or those who lived with at least two persons. This finding underlines a further need to promote online registration as a medical facility management tool. Moreover, potential barriers to accessing online registrations should be identified.

A growing number of medical facilities and diagnostic laboratories offer online access to test results. In this study, good socioeconomic status was significantly associated only with online checking of the results of medical/laboratory tests. This observation may result from the fact that laboratory tests in Poland are mostly offered by private companies that have developed their own IT systems dedicated to customer traffic management. The ability to check the results online reduces the number of patient contacts with the facility and is a cost-effective management solution.

Online pharmacies and medical stores in Poland offer a variety of services, including mail-order sales of over-the-counter drugs and dietary supplements, the possibility of booking a prescription drug in a pharmacy, and the possibility of checking the availability of the drug in a selected pharmacy [40]. Findings from this study showed that a significant percentage of Internet users in Poland (43.5%) purchase drugs or dietary supplements via the Internet. This finding underlines the high potential of the medical e-commerce market in Poland. Females, those with higher education, those who lived in cities above 100,000 residents, and those who lived with at least two persons were more likely to order drugs or dietary supplements online, which is in line with previous findings on the use of the Internet for health purposes [26,27]. The lack of age differences in the use of the Internet for drugs/dietary supplement purchases indicates that online pharmacies and medical stores may have a high potential for development, especially among older adults with chronic diseases.

Earlier studies carried out between 2013 and 2015 consistently showed that using the Internet for accessing health-related information in Poland correlated with younger age, and that people aged 50 years or more relatively rarely used the Internet as a source of health-related information [26,41]. In this study, people aged 50 years and over were less likely to search for information about doctors and medical services, search for a medical facility, or check online reviews of doctors. However, there were no age differences in the use of the Internet for accessing information on drugs and their effects, drug ordering, prescription orders, or checking health-related websites. This observation may result from the fact that social attitudes towards the use of the Internet for health purposes may change and the growing number of older adults are familiar with Internet websites and mobile devices.

This study has practical implications for policymakers and public health professionals in Poland. First, the most up-to-date characteristic of the use of the Internet for health purposes was presented. Second, factors associated with the use of the Internet for health purposes presented in this study may be used by policymakers to reduce the barriers to accessing the medical Internet. Third, a relatively high public support for the use of the Internet for health purposes presented in this study may encourage public health professionals to develop health promotion activities based on the web and eHealth. Moreover, data presented in this study may be used by healthcare providers to independently further development goals that will be based on eHealth services well-perceived by the public. Due to the relatively high adoption of medical Internet use, the public government should remove barriers to medical Internet access caused by the digital divide. As this study used a nationwide sample of adults in Poland, further studies should be carried out among patients with chronic diseases as well as vulnerable populations. Individual health status, long-term illnesses, and disabilities may also influence the use of the Internet for health purposes; therefore, further studies on the use of the Internet for health purposes in particular groups (patients with chronic diseases) should be carried out. Moreover, further studies should also analyze the impact of patterns of Internet use and differences in intensities of Internet use on the Internet use for health purposes.

There are several limitations of this study. This study was carried out using a web-based interview; therefore, the study population is limited to Internet users in Poland. Moreover, drawbacks of the cross-sectional study design should be considered. Nevertheless, a nationwide random-quota sample was used. The data source was self-reported responses; therefore, recall bias cannot be excluded. Moreover, in this study, only the 10 most popular reasons for the use of the Internet for health purposes were included. Moreover, only socioeconomic factors were included and questions on lifestyle factors (e.g., substance use) were not addressed.

## 5. Conclusions

This study confirmed a high level of adoption of medical Internet in Poland. Searching for health information, checking the drugs and their effects, as well as searching for doctors and medical facilities were the most common reasons for the use of the Internet for health purposes and indicates the high role of private institutions in the development of medical Internet in Poland. Female gender, having higher education, and living in cities from 100,000 to 499,999 residents were the most important factors associated with the use of the Internet for health purposes among adults in Poland. There were no economic barriers to medical Internet access; therefore, online public health interventions may be considered as a potential communication channel. Findings from this study may be used by policymakers and public health professionals to develop eHealth services tailored to individual socio-economic groups.

## Figures and Tables

**Table 1:** Characteristics of the study population (n = 1092).

Variable	n	%
Gender		
male	518	47.4
female	574	52.6
Age (years)		
18–29	242	22.2
30–39	232	21.2
40–49	176	16.1
50–59	190	17.4
60+	252	23.1
Educational level		
higher	471	43.1
less than higher	621	56.9
Marital status		
single	330	30.2
informal relationship	192	17.6
married	543	49.7
divorced or widowed	27	2.5
Having children		
yes	675	61.8
no	417	38.2
Place of residence		
rural	411	37.6
city < 20,000 residents	141	12.9
city 20,000–99,999 residents	210	19.2
city 100,000–499,999 residents	194	17.8
city ≥ 500,000 residents	136	12.5
Number of household members		
1	155	14.2
2	358	32.8
3 or more	579	53.0
Employment status		
currently employed/self-employed	674	61.7
unemployed, retired, or student	418	38.3
Self-declared economic status		
good	449	41.1
moderate	419	38.4
bad	224	20.5

**Table 2:** The use of the Internet for health purposes among adults in Poland (n = 1092).

	Use of the Internet for Health Purposes—Percentage of Respondents Who Answered “Yes” by Socioeconomic Factors									
Variable	Searching for Health Information		Searching for Information about Doctors and Medical Services		Searching for a Medical Facility		Checking Online Reviews of Doctors		Checking Online Reviews of Medical Facility	
	n (%)	*p*	n (%)	*p*	n (%)	*p*	n (%)	*p*	n (%)	*p*
Overall	709 (64.9)		692 (63.4)		713 (65.3)		603 (55.2)		562 (51.5)	
Gender										
male	298 (57.5)	<**0.001**	302 (58.3)	<**0.001**	315 (60.8)	**0.003**	247 (47.7)	<**0.001**	241 (46.5)	**0.002**
female	411 (71.6)		390 (67.9)		398 (69.3)		356 (62.0)		321 (55.9)	
Age (years)										
18–29	159 (65.7)	0.5	158 (65.3)	**0.01**	155 (64.0)	**0.003**	142 (58.7)	<**0.001**	130 (53.7)	**0.002**
30–39	159 (68.5)		155 (66.8)		166 (71.6)		147 (63.4)		142 (61.2)	
40–49	117 (66.5)		125 (71.0)		128 (72.7)		110 (62.5)		90 (51.1)	
50–59	117 (61.6)		115 (60.5)		119 (62.6)		87 (45.8)		92 (48.4)	
60+	157 (62.3)		139 (55.2)		145 (57.5)		117 (46.4)		108 (42.9)	
Educational level										
higher	328 (69.6)	**0.004**	327 (69.4)	<**0.001**	337 (71.5)	<**0.001**	293 (62.2)	<**0.001**	262 (55.6)	**0.02**
less than higher	381 (61.4)		365 (58.8)		376 (60.5)		310 (49.9)		300 (48.3)	
Marital status										
single	196 (59.4)	0.07	200 (60.6)	0.5	195 (59.1)	**0.03**	172 (52.1)	0.4	158 (47.9)	0.3
informal relationship	132 (68.8)		121 (63.0)		125 (65.1)		107 (55.7)		104 (54.2)	
married	361 (66.5)		355 (65.4)		375 (69.1)		311 (57.3)		288 (53.0)	
divorced or widowed	20 (74.1)		16 (59.3)		18 (66.7)		13 (48.1)		12 (44.4)	
Having children										
yes	456 (67.6)	**0.02**	440 (65.2)	0.1	455 (67.4)	0.06	379 (56.1)	0.4	361 (53.5)	0.09
no	253 (60.7)		252 (60.4)		258 (61.9)		224 (53.7)		201 (48.2)	
Place of residence										
rural	250 (60.8)	0.2	250 (60.8)	**0.003**	252 (61.3)	**0.004**	205 (49.9)	**0.005**	190 (46.2)	**0.002**
city < 20,000 residents	90 (63.8)		76 (53.9)		81 (57.4)		74 (52.5)		62 (44.0)	
city 20,000–99,999 residents	143 (68.1)		135 (64.3)		142 (67.6)		121 (57.6)		123 (58.6)	
city 100,000–499,999 residents	134 (69.1)		143 (73.7)		145 (74.7)		128 (66.0)		115 (59.3)	
city ≥ 500,000 residents	92 (67.6)		88 (64.7)		93 (68.4)		75 (55.1)		72 (52.9)	
Number of household members										
1	92 (59.4)	0.09	91 (58.7)	0.07	94 (60.6)	0.05	82 (52.9)	**0.03**	69 (44.5)	**0.002**
2	225 (62.8)		216 (60.3)		222 (62.0)		180 (50.3)		166 (46.4)	
3 or more	392 (67.7)		385 (66.5)		397 (68.6)		341 (58.9)		327 (56.5)	
Employment status										
currently employed/self-employed	440 (65.3)	0.8	442 (65.6)	0.05	460 (68.2)	**0.01**	386 (57.3)	0.08	365 (54.2)	**0.02**
unemployed, retired, or student	269 (64.4)		250 (59.8)		253 (60.5)		217 (51.9)		197 (47.1)	
Self-declared economic status										
good	296 (65.9)	0.3	291 (64.8)	0.2	304 (67.7)	0.2	261 (58.1)	0.2	247 (55.0)	0.1
moderate	278 (66.3)		270 (64.4)		273 (65.2)		227 (54.2)		204 (48.7)	
bad	135 (60.3)		131 (58.5)		136 (60.7)		115 (51.3)		111 (49.6)	
	**Use of the Internet for Health Purposes—Percentage of Respondents Who Answered “Yes” by Socioeconomic Factors**									
**Variable**	**Making an Appointment with a Doctor**		**Checking the Results of Medical/Laboratory Tests**		**Searching for Information on Drugs and Their Effects**		**Ordering a Prescription for Drugs via the Internet**		**Purchase of Drugs or Dietary Supplements via the Internet**	
	**n (%)**	***p***	**n (%)**	***p***	**n (%)**	***p***	**n (%)**	***p***	**n (%)**	***p***
Overall	478 (43.8)		568 (52.0)		763 (69.9)		303 (27.7)		475 (43.5)	
Gender										
male	209 (40.3)	**0.03**	254 (49.0)	0.06	317 (61.2)	<**0.001**	149 (28.8)	0.5	208 (40.2)	**0.03**
female	269 (46.9)		314 (54.7)		446 (77.7)		154 (26.8)		267 (46.5)	
Age (years)										
18–29	118 (48.8)	0.2	121 (50.0)	0.3	156 (64.5)	0.1	74 (30.6)	0.6	97 (40.1)	**0.01**
30–39	104 (44.8)		125 (53.9)		175 (75.4)		69 (29.7)		118 (50.9)	
40–49	82 (46.6)		101 (57.4)		121 (68.8)		47 (26.7)		86 (48.9)	
50–59	76 (40.0)		102 (53.7)		131 (68.9)		46 (24.2)		81 (42.6)	
60+	98 (38.9)		119 (47.2)		180 (71.4)		67 (26.6)		93 (36.9)	
Educational level										
higher	243 (51.6)	<**0.001**	289 (61.4)	<**0.001**	357 (75.8)	<**0.001**	148 (31.4)	**0.02**	242 (51.4)	<**0.001**
less than higher	235 (37.8)		279 (44.9)		406 (65.4)		155 (25.0)		233 (37.5)	
Marital status										
single	124 (37.6)	0.06	148 (44.8)	**0.004**	208 (63.0)	**0.01**	90 (27.3)	0.6	136 (41.2)	0.8
informal relationship	90 (46.9)		96 (50.0)		140 (72.9)		61 (31.8)		86 (44.8)	
married	252 (46.4)		311 (57.3)		393 (72.4)		144 (26.5)		240 (44.2)	
divorced or widowed	12 (44.4)		13 (48.1)		22 (81.5)		8 (29.6)		13 (48.1)	
Having children										
yes	308 (45.6)	0.1	378 (56.0)	<**0.001**	499 (73.9)	<**0.001**	191 (28.3)	0.6	294 (43.6)	0.9
no	170 (40.8)		190 (45.6)		264 (63.3)		112 (26.9)		181 (43.4)	
Place of residence										
rural	156 (38.0)	<**0.001**	194 (47.2)	**0.02**	263 (64.0)	**0.002**	97 (23.6)	**0.004**	154 (37.5)	**0.002**
city < 20,000 residents	51 (36.2)		66 (46.8)		97 (68.8)		29 (20.6)		57 (40.4)	
city 20,000–99,999 residents	87 (41.4)		114 (54.3)		146 (69.5)		63 (30.0)		92 (43.8)	
city 100,000–499,999 residents	106 (54.6)		114 (58.8)		154 (79.4)		69 (35.6)		100 (51.5)	
city ≥ 500,000 residents	78 (57.4)		80 (58.8)		103 (75.7)		45 (33.1)		72 (52.9)	
Number of household members										
1	55 (35.5)	**0.03**	61 (39.4)	<**0.001**	99 (63.9)	0.2	43 (27.7)	0.2	66 (42.6)	**0.001**
2	152 (42.5)		175 (48.9)		254 (70.9)		87 (24.3)		129 (36.0)	
3 or more	271 (46.8)		332 (57.3)		410 (70.8)		173 (29.9)		280 (48.4)	
Employment status										
currently employed/self-employed	315 (46.7)	**0.01**	379 (56.2)	<**0.001**	469 (69.6)	0.8	198 (29.4)	0.1	320 (47.5)	<**0.001**
unemployed, retired, or student	163 (39.0)		189 (45.2)		294 (70.3)		105 (25.1)		155 (37.1)	
Self-declared economic status										
good	202 (45.0)	0.6	254 (56.6)	**0.02**	314 (69.9)	0.7	124 (27.6)	0.8	194 (43.2)	0.4
moderate	175 (41.8)		213 (50.8)		297 (70.9)		113 (27.0)		191 (45.6)	
bad	101 (45.1)		101 (45.1)		152 (67.9)		66 (29.5)		90 (40.2)	

Statistically significant values are bolded.

**Table 3:** Factors associated with the use of the Internet for health purposes (n = 1092).

	Factors Associated with the Use of the Internet for Health Purposes—Multivariable Logistic Regression Model									
Variable	Searching for Health Information		Searching for Information about Doctors and Medical Services		Searching for a Medical Facility		Checking Online Reviews of Doctors		Checking Online Reviews of Medical Facility	
	OR (95% CI)	*p*	OR (95% CI)	*p*	OR (95% CI)	*p*	OR (95% CI)	*p*	OR (95% CI)	*p*
Gender										
male	Reference	<**0.001**	Reference		Reference		Reference		Reference	
female	1.82 (1.39–2.37)		1.47 (1.13–1.92)	**0.004**	1.47 (1.12–1.92)	**0.005**	1.83 (1.41–2.37)	<**0.001**	1.46 (1.13–1.89)	**0.003**
Age (years)										
18–29	1.55 (0.90–2.66)	0.1	2.29 (1.34–3.91)	**0.002**	1.87 (1.09–3.20)	**0.02**	2.31 (1.37–3.92)	**0.002**	1.90 (1.13–3.20)	**0.02**
30–39	1.51 (0.91–2.49)	0.1	1.91 (1.17–3.12)	**0.01**	2.00 (1.21–3.29)	**0.01**	2.24 (1.38–3.64)	**0.001**	2.13 (1.32–3.45)	**0.002**
40–49	1.22 (0.74–2.01)	0.4	2.18 (1.32–3.61)	**0.003**	1.97 (1.18–3.28)	**0.01**	2.02 (1.24–3.30)	**0.005**	1.26 (0.78–2.03)	0.4
50–59	0.96 (0.61–1.51)	0.9	1.32 (0.84–2.06)	0.2	1.21 (0.77–1.89)	0.4	0.97 (0.62–1.50)	0.9	1.12 (0.72–1.74)	0.6
60+	Reference		Reference		Reference		Reference		Reference	
Educational level										
higher	1.38 (1.06–1.81)	**0.02**	1.55 (1.18–2.03)	**0.002**	1.49 (1.14–1.96)	**0.004**	1.52 (1.17–1.98)	**0.002**	1.23 (0.95–1.60)	0.1
less than higher	Reference		Reference		Reference		Reference		Reference	
Marital status										
ever married	1.04 (0.73–1.48)	0.8	1.13 (0.80–1.60)	0.5	1.50 (1.05–2.13)	**0.03**	1.33 (0.94–1.87)	0.1	1.01 (0.72–1.41)	0.9
never married	Reference		Reference		Reference		Reference		Reference	
Having children										
yes	1.47 (0.98–2.23)	0.07	1.51 (1.01–2.28)	**0.04**	1.27 (0.84–1.92)	0.3	1.29 (0.86–1.92)	0.2	1.57 (1.05–2.34)	**0.03**
no	Reference		Reference		Reference		Reference		Reference	
Place of residence										
rural	Reference		Reference		Reference		Reference		Reference	
city < 20,000 residents	1.14 (0.76–1.71)	0.5	0.71 (0.48–1.06)	0.09	0.82 (0.55–1.22)	0.3	1.08 (0.73–1.61)	0.7	0.92 (0.62–1.37)	0.7
city 20,000–99,999 residents	1.39 (0.97–2.01)	0.08	1.12 (0.78–1.61)	0.5	1.30 (0.90–1.87)	0.2	1.36 (0.96–1.94)	0.09	1.67 (1.17–2.37)	**0.004**
city 100,000–499,999 residents	1.49 (1.02–2.18)	**0.04**	1.82 (1.23–2.70)	**0.003**	1.96 (1.31–2.91)	<**0.001**	2.05 (1.41–2.99)	<**0.001**	1.85 (1.28–2.66)	<**0.001**
city ≥ 500,000 residents	1.39 (0.90–2.15)	0.1	1.16 (0.76–1.78)	0.5	1.37 (0.89–2.12)	0.2	1.21 (0.80–1.83)	0.4	1.42 (0.94–2.15)	0.09
Number of household members										
1	Reference		Reference		Reference		Reference		Reference	
2	1.20 (0.78–1.83)	0.4	1.05 (0.69–1.62)	0.8	0.95 (0.62–1.47)	0.8	0.88 (0.58–1.34)	0.5	1.12 (0.74–1.71)	0.6
3 or more	1.41 (0.90–2.19)	0.1	1.15 (0.74–1.79)	0.5	1.12 (0.72–1.75)	0.6	1.06 (0.69–1.64)	0.8	1.47 (0.95–2.27)	0.08
Employment status										
currently employed/self-employed	0.95 (0.70–1.30)	0.8	0.99 (0.73–1.34)	0.9	1.09 (0.80–1.48)	0.6	0.98 (0.72–1.32)	0.9	1.10 (0.82–1.48)	0.5
unemployed, retired, or student	Reference		Reference		Reference		Reference		Reference	
Self-declared economic status										
good	1.23 (0.87–1.74)	0.3	1.26 (0.89–1.79)	0.2	1.26 (0.88–1.79)	0.2	1.24 (0.88–1.74)	0.2	1.17 (0.83–1.64)	0.4
moderate	1.32 (0.93–1.86)	0.1	1.26 (0.90–1.79)	0.2	1.16 (0.82–1.64)	0.4	1.09 (0.77–1.53)	0.6	0.95 (0.68–1.33)	0.8
bad	Reference		Reference		Reference		Reference		Reference	
	**Factors Associated with the Use of the Internet for Health Purposes—Multivariable Logistic Regression Model**									
**Variable**	**Making an Appointment with a Doctor**		**Checking the Results of Medical/Laboratory Tests**		**Searching for Information on Drugs and Their Effects**		**Ordering a Prescription for Drugs via the Internet**		**Purchase of Drugs or Dietary Supplements via the Internet**	
	**OR (95% CI)**	***p***	**OR (95% CI)**	***p***	**OR (95% CI)**	***p***	**OR (95% CI)**	***p***	**OR (95% CI)**	***p***
Gender										
male	Reference		Reference		Reference		Reference		Reference	
female	1.28 (0.99–1.66)	0.06	1.27 (0.98–1.64)	0.07	2.11 (1.60–2.80)	<**0.001**	0.88 (0.66–1.16)	0.4	1.41 (1.09–1.82)	**0.01**
Age (years)										
18–29	2.09 (1.23–3.54)	**0.01**	1.22 (0.72–2.06)	0.5	1.04 (0.59–1.83)	0.9	1.22 (0.69–2.14)	0.5	0.72 (0.43–1.22)	0.2
30–39	1.25 (0.77–2.02)	0.4	1.02 (0.63–1.65)	0.9	1.59 (0.92–2.72)	0.1	0.99 (0.59–1.67)	0.9	1.02 (0.63–1.65)	0.9
40–49	1.22 (0.75–1.98)	0.4	1.10 (0.67–1.79)	0.7	0.93 (0.55–1.58)	0.8	0.83 (0.49–1.41)	0.5	0.97 (0.60–1.58)	0.9
50–59	0.96 (0.61–1.50)	0.8	1.03 (0.66–1.61)	0.9	0.91 (0.56–1.47)	0.7	0.78 (0.47–1.28)	0.3	0.93 (0.59–1.45)	0.7
60+	Reference		Reference		Reference		Reference		Reference	
Educational level										
higher	1.68 (1.30–2.18)	<**0.001**	1.86 (1.43–2.41)	<**0.001**	1.53 (1.14–2.03)	**0.004**	1.33 (1.01–1.77)	**0.04**	1.58 (1.22–2.05)	<**0.001**
less than higher	Reference		Reference		Reference		Reference		Reference	
Marital status										
ever married	1.26 (0.90–1.78)	0.2	1.20 (0.85–1.67)	0.3	0.99 (0.68–1.44)	0.9	0.83 (0.58–1.20)	0.3	1.18 (0.84–1.65)	0.3
never married	Reference		Reference		Reference		Reference		Reference	
Having children										
yes	1.43 (0.96–2.15)	0.08	1.41 (0.95–2.10)	0.09	1.60 (1.03–2.48)	**0.04**	1.40 (0.91–2.18)	0.1	0.74 (0.50–1.10)	0.1
no	Reference		Reference		Reference		Reference		Reference	
Place of residence										
rural	Reference		Reference		Reference		Reference		Reference	
city < 20,000 residents	0.88 (0.58–1.32)	0.5	0.94 (0.63–1.40)	0.8	1.27 (0.83–1.94)	0.3	0.84 (0.53–1.35)	0.5	1.14 (0.76–1.70)	0.5
city 20,000–99,999 residents	1.12 (0.79–1.60)	0.5	1.30 (0.91–1.84)	0.2	1.30 (0.89–1.88)	0.2	1.40 (0.95–2.05)	0.09	1.35 (0.95–1.92)	0.1
city 100,000–499,999 residents	2.05 (1.43–2.95)	<**0.001**	1.70 (1.18–2.46)	**0.005**	2.30 (1.51–3.52)	<**0.001**	1.82 (1.24–2.68)	**0.002**	1.89 (1.31–2.72)	<**0.001**
city ≥ 500,000 residents	2.32 (1.53–3.53)	<**0.001**	1.66 (1.09–2.52)	**0.02**	1.84 (1.15–2.95)	**0.01**	1.63 (1.05–2.54)	**0.03**	1.88 (1.24–2.84)	**0.003**
Number of household members										
1	Reference		Reference		Reference		Reference		Reference	
2	1.38 (0.89–2.13)	0.1	1.39 (0.91–2.14)	0.1	1.57 (0.99–2.46)	0.05	0.95 (0.60–1.51)	0.8	0.85 (0.55–1.29)	0.4
3 or more	1.58 (1.01–2.48)	**0.04**	2.03 (1.30–3.16)	**0.002**	1.64 (1.03–2.61)	**0.04**	1.28 (0.80–2.06)	0.3	1.68 (1.08–2.60)	**0.02**
Employment status										
currently employed/self-employed	1.21 (0.90–1.63)	0.2	1.36 (1.01–1.83)	**0.04**	0.93 (0.70–1.29)	0.7	1.18 (0.85–1.64)	0.3	1.33 (0.98–1.79)	0.07
unemployed, retired, or student	Reference		Reference		Reference		Reference		Reference	
Self-declared economic status										
good	0.85 (0.60–1.20)	0.3	1.42 (1.01–2.00)	**0.04**	1.07 (0.74–1.54)	0.7	0.86 (0.59–1.24)	0.4	1.01 (0.71–1.42)	0.9
moderate	0.80 (0.57–1.12)	0.2	1.22 (0.87–1.72)	0.3	1.19 (0.82–1.71)	0.4	0.87 (0.60–1.26)	0.5	1.20 (0.85–1.69)	0.3
bad	Reference		Reference		Reference		Reference		Reference	

Abbreviations: OR—odds ratio; 95% CI—95% confidence interval. Statistically significant values are bolded.

## Data Availability

Data are available upon reasonable request. The dataset used to conduct the analyses is available from corresponding author upon reasonable request.
